# Cost-Efficient Wafer-Level Capping for MEMS and Imaging Sensors by Adhesive Wafer Bonding

**DOI:** 10.3390/mi7100192

**Published:** 2016-10-18

**Authors:** Simon J. Bleiker, Maaike M. Visser Taklo, Nicolas Lietaer, Andreas Vogl, Thor Bakke, Frank Niklaus

**Affiliations:** 1 Department of Micro and Nanosystems, KTH Royal Institute of Technology, SE-100 44 Stockholm, Sweden; bleiker@kth.se; 2 Department of Instrumentation, SINTEF ICT, NO-0314 Oslo, Norway; maaike.taklo@sintef.no; 3 Department of Microsystems and Nanotechnology, SINTEF ICT, NO-0314 Oslo, Norway; nicolas.lietaer@sintef.no (N.L.); andreas.vogl@sintef.no (A.V.); thor.bakke@tunableir.com (T.B.)

**Keywords:** micro electro-mechanical systems (MEMS), imaging sensor, packaging, adhesive wafer bonding, benzocyclobutene (BCB)

## Abstract

Device encapsulation and packaging often constitutes a substantial part of the fabrication cost of micro electro-mechanical systems (MEMS) transducers and imaging sensor devices. In this paper, we propose a simple and cost-effective wafer-level capping method that utilizes a limited number of highly standardized process steps as well as low-cost materials. The proposed capping process is based on low-temperature adhesive wafer bonding, which ensures full complementary metal-oxide-semiconductor (CMOS) compatibility. All necessary fabrication steps for the wafer bonding, such as cavity formation and deposition of the adhesive, are performed on the capping substrate. The polymer adhesive is deposited by spray-coating on the capping wafer containing the cavities. Thus, no lithographic patterning of the polymer adhesive is needed, and material waste is minimized. Furthermore, this process does not require any additional fabrication steps on the device wafer, which lowers the process complexity and fabrication costs. We demonstrate the proposed capping method by packaging two different MEMS devices. The two MEMS devices include a vibration sensor and an acceleration switch, which employ two different electrical interconnection schemes. The experimental results show wafer-level capping with excellent bond quality due to the re-flow behavior of the polymer adhesive. No impediment to the functionality of the MEMS devices was observed, which indicates that the encapsulation does not introduce significant tensile nor compressive stresses. Thus, we present a highly versatile, robust, and cost-efficient capping method for components such as MEMS and imaging sensors.

## 1. Introduction

Facilitating the miniaturization of sensor and actuator components and their direct integration with conventional integrated circuits (ICs) is critical for emerging application areas, such as the internet of things (IoT) and wearable electronics. These applications frequently utilize components including micro electro-mechanical systems (MEMS) and imaging sensors that generally have to be capped to protect them from environmental influences such as dust or humidity. Encapsulation is typically one of the most expensive process steps in the entire device fabrication. An efficient and cost-effective capping process at the wafer-level is therefore crucial for the feasibility of a high-volume fabrication of MEMS and imaging components.

Existing capping methods typically rely on wafer bonding to join the device substrate with a capping substrate as a protection against influences from the environment. Wafer bonding has therefore become a key technology in the packaging of MEMS and imaging sensors [1,2]. Anodic bonding [3], eutectic bonding [4], and Si–Si direct bonding [5] are among the most prevalent bonding methods in MEMS fabrication. However, these bonding technologies typically involve large thermal budgets and/or high voltages, and they typically require extremely flat bonding surfaces with low surface roughness, which makes these techniques challenging for use in some MEMS and imaging sensor applications. Thermocompression bonding of gold or copper films [6,7,8] has been developed for hermetic encapsulation of vacuum cavities. Metallic thermocompression bonding is a versatile method for MEMS capping, although it typically requires a fairly large temperature budget to achieve high reliability. Adhesive wafer bonding [1,9] is a robust and low-cost process that is extensively used for capping and integration of optical sensors [10,11]. Various polymer adhesives that enable fairly low bonding temperatures and exceptional mechanical and chemical robustness have been developed. Most adhesives are transparent, which makes this bonding method well-suited for optical sensors and very cost-sensitive MEMS applications that do not require a fully hermetic package [9,12].

However, capping MEMS and optical devices without impeding their functionality typically requires selective adhesive bonding. Selective adhesive bonding describes a process in which only certain areas of the device wafer and the capping wafer are bonded, while other areas are left unbonded. This enables the encapsulation of MEMS and optical devices in sealed cavities, thus protecting them from environmental influences. Various approaches to selective adhesive bonding have been presented in the literature. Some of the more specialized approaches include localized laser heating [13], localization of UV-curable adhesive by centrifugal spinning [14], and transfer bonding of pre-formed benzocyclobutene (BCB) caps [15]. More common approaches to selective adhesive bonding are based on patterning of photosensitive adhesives by photolithography, or masking and etching of non-photosensitive adhesives [12,16,17,18]. However, patterning by either photolithography or etching typically requires partial cross-linking of the polymer adhesive, for example by baking. Partial cross-linking before bonding lowers the resulting bond strength and bonding yield due to reduced re-flow behavior of the polymer adhesive [18]. This creates a stringent trade-off between the capability of patterning the adhesive layer and the resulting bond strength; insufficient cross-linking renders the patterning of the polymer adhesive impossible, while excessive cross-linking compromises the bonding capability of the polymer adhesive. The result is a narrow process window, and thus a reduced robustness of the process. Other reported methods for the creation of patterned adhesive layers are local dispensing, screen printing, and stamp-printing [9,19,20]. Dispensing and printing methods often suffer from poor layer thickness control, imprecise alignment, and limited resolution of the patterned adhesive layer. Furthermore, most of the mentioned capping methods using selective adhesive bonding substantially increase the process complexity and therefore result in increased fabrication costs.

In this paper, we propose an extremely simple, robust, and cost-effective fabrication process for wafer-level capping of MEMS devices and imaging sensors by adhesive wafer bonding. In this process, a capping substrate with etched cavities covered by a thin layer of spray-coated polymer adhesive is bonded to the device substrate. No patterning of the adhesive is necessary. This ensures a uniform and pristine adhesive layer, which is otherwise not achievable with selective adhesive bonding approaches. Therefore, the proposed capping process provides excellent bonding yield and reduces the complexity and cost of the process. We demonstrate the capping process for two different MEMS devices: a vibration sensor and an acceleration switch. For these demonstrat ions, the thermosetting polymer benzocyclobutene (BCB) was used. BCB has a low bonding temperature of <250 °C which makes the proposed capping method fully compatible with complementary metal-oxide-semiconductor (CMOS) circuits.

## 2. Wafer-Level Capping Method

**Figure 1:**
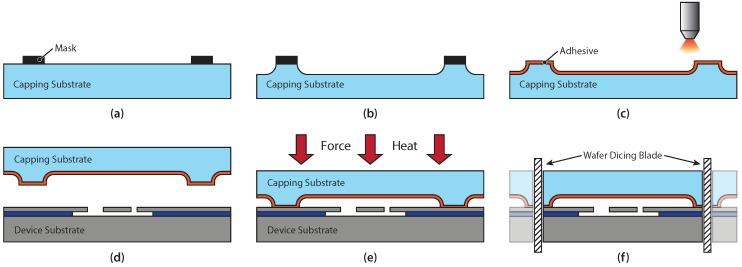
Schematic process flow of the proposed capping method. (**a**) Patterning of a mask on the capping substrate; (**b**) Etching of the cavities and stripping of the mask; (**c**) Deposition of a thin layer of polymer adhesive by spray-coating; (**d**) Alignment of the capping substrate and the device substrate and moving them in contact; (**e**) Adhesive wafer bonding by applying a force to the wafer stack and raising the temperature to cure the polymer adhesive; (**f**) Die singulation by wafer dicing.

Our proposed capping method follows three basic fabrication steps, outlined in Figure 1: first, preparation of the capping substrate and spray-coating of the adhesive polymer layer; second, adhesive wafer bonding; and last, die singulation to separate the wafer into single chips. The preparation of the capping substrate (shown in Figure 1a–c) consists of patterning a mask, etching cavities, and applying a thin layer of polymer adhesive by spray-coating. In Figure 1d,e, the capping substrate is aligned to a fully processed device substrate, containing the MEMS devices or imaging sensors, and subsequently bonded by applying the bonding force and heat to cure the polymer adhesive. As a final step, the devices are separated into individual chips by conventional wafer dicing, as indicated in Figure 1f.

The capping process is designed such that the substrate preparation, which comprises the cavity formation and adhesive deposition, is performed entirely on the capping substrate. Therefore, no additional processing or preparation steps on the device substrate are required after the completed fabrication of the device to be capped. The simplicity and versatility of this capping method make it potentially interesting for a large number of MEMS and optical device applications.

In the following sections, we demonstrate the proposed method for the encapsulation of two different MEMS devices. For both demonstrations, BCB is used as the intermediate adhesive layer. However, the two different demonstrator devices are implemented utilizing two different electrical interconnection concepts, as discussed in Section 2.3.

### 2.1. Materials for Demonstrator Device Fabrication

The capping process, as well as the choice of substrates and materials involved are specifically designed to be as simple, robust, and cost-effective as possible. For the two demonstrator devices, Borofloat 33 glass substrates were chosen for the capping wafers. Glass substrates are available at comparably low cost and can be easily processed by using, for example, hydrofluoric acid (HF) etching. Due to their transparent nature, glass substrates are also suitable as capping wafers for imaging sensors. In addition, glass capping substrates allow for visual inspection of the quality of the bond interface after the device encapsulation.

BCB (Cyclotene${}^{\text{®}}$ 3022-35, The Dow Chemical Company, Midland, MI, USA) was chosen as intermediate polymer adhesive due to its excellent mechanical stability, chemical inertness, and low curing temperature of $250{\;}^{\circ }C$. BCB is highly transparent in the visible wavelength spectrum, with an optical transmittance of >99.63% for wavelengths above 380nm [21]. Furthermore, spray-coated and uncured BCB exhibits excellent re-flow capabilities and can easily compensate for wafer surface topographies during the wafer bonding step. During the curing process, BCB does not release any outgassing by-products, which lowers the risk of void formation and delamination at the bond interface [9,22]. After curing, BCB features a glass transition temperature of >350 ${}^{\circ }C$ and a very low moisture uptake of <0.2%, which ensures an excellent compatibility with a large variety of post-bonding processes. BCB is commercially available and widely used in the electronics and semiconductor industry.

BCB can be deposited at the wafer-level by spin-coating or spray-coating. Spin-coating offers a higher level of thickness control and uniformity than spray-coating. Thickness uniformity is, however, not crucial thanks to the re-flow ability of BCB. Spray-coating, on the other hand, uses smaller volumes of adhesive due to smaller material losses in the deposition process, and enables uniform coating of substrates with high surface topographies. Therefore, spray-coating was chosen to deposit the BCB on the capping substrate in order to minimize the material losses and optimize the cost-effectiveness of the presented capping method.

### 2.2. Fabrication Process for Demonstrator Devices

For both demonstrator devices, the preparation of the glass capping substrate starts with patterning a metal hard mask, defining the cavities by standard photolithography and metal etching, as shown in Figure 1a. Metal combinations such as NiCr/Au or TiW/Au are suitable materials for the mask. The cavities are etched in 49% HF, which provides an etch rate of around 7μm/min, as illustrated in Figure 1b. The depth of the cavities can be easily adjusted in the range from a few hundred nmup to hundreds of μm, depending on the requirements of the specific application. The deposition of the BCB adhesive by spray-coating is depicted in Figure 1c. The uncured (i.e., not cross-linked) BCB used in this process has the ability to compensate for topography and particles due to its re-flow property, as long as the BCB layer thickness is larger than the topography or particle size. In this work, BCB layer thicknesses of 1.4μm and 3μm were used, which were achieved by manual spray-coating of diluted BCB with solvent ratios between 1:1 and 1:3 (T1100 rinse solvent, The Dow Chemical Company). The capping wafer is then baked on a hotplate at $110{\;}^{\circ }C$ for 90s to remove the solvents from the spray-coated BCB layer. It should be noted that this step does not initiate the cross-linking of the BCB polymer. Therefore, the BCB retains its full re-flow capability.

After the preparation of the capping substrate, both substrates are aligned and put into contact using a BA6 wafer bond aligner (Suss Microtec, Munich, Germany), as indicated in Figure 1d. The device substrate contains fully fabricated MEMS structures, and no further preparations are required on the device substrate before the wafer bonding step. The bonding step, shown in Figure 1e, is then performed in a Suss Microtec SB6 bonder. To reach the desired bonding condition, the chamber is evacuated to <500 mbar, and the wafers are pre-heated to $150\;{}^{\circ }C$ for 5min for dehydration. Next, a bond force of 2.9kN is applied, and the wafers are heated to a temperature of $250\;{}^{\circ }C$ for 1h, which completely cures the BCB. The total bond area is ∼4440 ${\mathrm{mm}}^{2}$ for the acceleration switch wafer and ∼5502 ${\mathrm{mm}}^{2}$ for the vibration sensor wafer, thus resulting in an effective bonding pressure of 650kPa and 525kPa, respectively. The bonded substrates are allowed to cool down inside the chamber prior to the extraction from the wafer bonder. As the final step after the bonding, the chips are separated by wafer dicing, as depicted in Figure 1f.

### 2.3. Interconnection and Packaging Concepts

Most MEMS devices and imaging sensors require electrical connections from the inside of the encapsulated chip to the outside world. The electrical connection from within the encapsulation to a connection pad on the outside of the package can be established in two ways; either horizontal feed-throughs can be made that cross the bonded area, or alternatively, vertical through-silicon vias (TSVs) can be made that connect to a pad on the back-side of the substrate, thus avoiding crossing of the bonded area. The connection pads on the front or back-side of the chip are then coupled to the outside package by different integration schemes. Two of the most common integration schemes for chip-to-chip and chip-to-package interconnection are wire bonding and flip-chip bonding. Wire bonding is able to connect chips that are placed side-by-side or stacked on top of each other with an offset to reveal the underlying bond pads. Flip-chip bonding is based on vertical interconnection of stacked chips, which offers more compact integration and shorter signal lines; however, it typically increases the fabrication costs [2].

**Figure 2:**
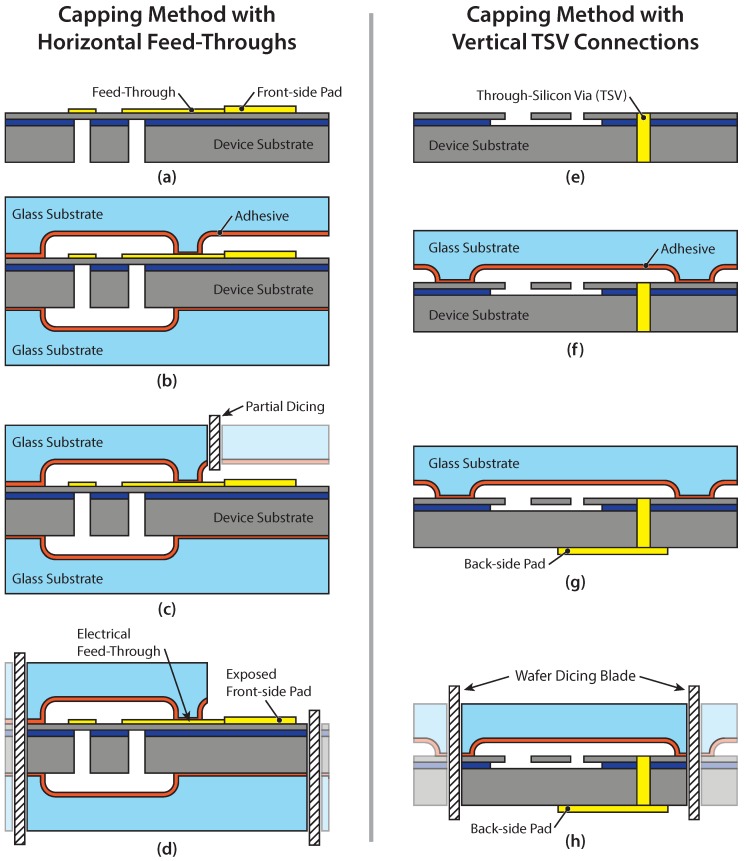
Capping process demonstrated for two different electrical interconnection schemes. (**a**–**d**) Front and back-side capping with horizontal electrical feed-throughs. Partial dicing is used to reveal the pads. Suitable, for example, for wire bond integration; (**e**–**h**) Front-side capping with through-silicon vias (TSVs) providing a vertical connection through the device substrate. Suitable, for example, for flip-chip integration.

The presented capping method is compatible with both horizontal feed-throughs as well as vertical TSV connections, which is demonstrated in this paper by the integration and experimental verification of two different MEMS devices in fully functional packages. The capping method with horizontal feed-throughs is demonstrated by utilizing wire bonding integration, while the capping method with vertical TSVs is demonstrated by employing flip-chip integration [22,23]. A detailed schematic of the two interconnection approaches is depicted in a side-by-side comparison in Figure 2. In both cases, the fabrication starts by establishing the electrical through-connections. The horizontal feed-through approach in Figure 2a–d shows the deposition of a surface feed-through and front-side pad for later wire bonding in step (a). Next, the glass capping substrate is bonded to the device wafer, as indicated in Figure 2b. This process step highlights the capability of capping the device substrate both on the front and back side, if required. To compensate for the topography of the surface feed-throughs passing through the bonded area, the chosen adhesive layer thickness has to be thicker than the metal layer. The front-side pad is then revealed by partial dicing that reaches just deep enough to cut through the glass lid, as illustrated in Figure 2c. Due to the partial dicing, the surface feed-through approach requires deeper cavities on the order of 60 to 80μm to ensure a sufficient margin for the precision of the dicing depth to avoid damaging the device substrate. The completed wafer is then separated into chips by wafer dicing, as shown in Figure 2d.

The vertical TSV approach in Figure 2e–h starts with the fabrication of the TSVs going through the entire device substrate, as depicted in Figure 2e. The TSV fabrication is performed either before or after the MEMS fabrication, depending on the type of TSV used. Next, the glass capping wafer is bonded to the front-side of the device wafer, as shown in Figure 2f. The back-side pads (depicted in Figure 2g) are simply deposited on the back side of the substrate after the bonding step. Wafer dicing is employed to separate the completed wafer into individual chips, as indicated in Figure 2h. Finally, the system integration of both the horizontal feed-through and the TSV capping approach is completed by connecting the chips to the package by wire bonding or flip-chip bonding, respectively.

## 3. Results and Discussion

### 3.1. Capping Results by Adhesive Wafer Bonding Using Spray-Coated BCB

**Figure 3:**
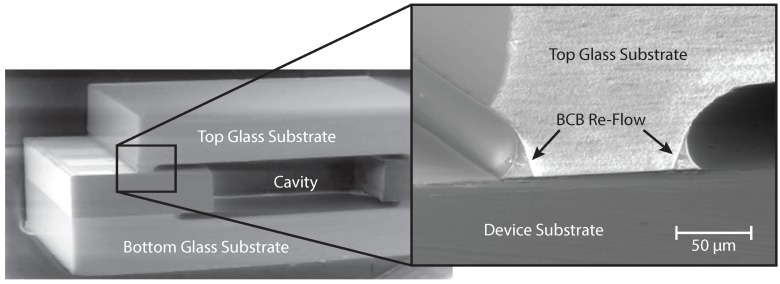
Scanning electron microscopy (SEM) cross-section of a front and back-side capped cavity, including partial dicing. No defects were found at the bond interface. The inset shows the re-flow behavior of the benzocyclobutene (BCB), which causes the accumulation of excess BCB at the edges of the cavities. Originally published in the Proceedings of the Pan Pacific Microelectronics Symposium, Kauai, HI, USA, 22–24 January 2008.

Bonded samples consisting of silicon substrates with front and back-side glass caps were used to evaluate the bond quality of the capping process using adhesive wafer bonding with spray-coated BCB as an intermediate adhesive layer. The samples contained encapsulated cavities as well as front-side pads to verify the partial dicing process. The quality of the adhesive bond was examined both visually through the transparent glass lid as well as by scanning electron microscopy (SEM) imaging of cross-sections of the bond interface. In Figure 3, a cross-section of a sealed cavity is presented, including a close-up of the bond interface. The optical inspection revealed an excellent bond quality without any voids or discontinuities. Thus, the bonded samples can withstand manual handling and wafer dicing without difficulty.

The re-flow behavior of BCB is clearly visible in the inset of Figure 3. Due to the low viscosity of BCB during the curing step, most of the BCB is squeezed out from the contact interface between the glass cap and the device wafer. The excess BCB gathers at the edges of the cavities in the glass cap, which further increases the bond strength. This effect has to be taken into account in the design of the capping wafer, in order to leave a sufficient distance between the device and the edge of the cavity. Additionally, the BCB layer thickness influences the quantity of excess BCB that accumulates at the edges of the cavities. The accumulation of BCB can be reduced by pre-curing, and thus partially cross-linking the BCB before the bonding step, which limits the re-flow capability of the polymer. This may result in an improved control of the wafer alignment, the bond layer thickness, and thus the substrate separation [24,25]. However, it comes at the price of an increased risk of void formation at the bond interface, due to the diminished capability to compensate for surface topography [24].

Single-side bonded samples with a glass cap on only the front-side of the substrate were fabricated to characterize the bond strength of the presented method. A Dage 2400A shear tester with a 50kgf load cartridge was used to measure the bond strength of single dies. The shear tester was operated at a speed of 17μm/s and a test height of 175μm. The test revealed consistent bond strength values of >20–30 MPa, which is well above the minimum requirement defined by the MIL-STD-883H standard (6MPa) [26].

### 3.2. Demonstrator Devices

Two MEMS demonstrator devices were developed and fabricated to study the feasibility and reliability of the proposed capping method. The two demonstrator devices include a vibration sensor with horizontal feed-through interconnections, and an acceleration switch with vertical TSV connections. Both devices feature Borofloat 33 glass capping substrates that were bonded to silicon device substrates with spray-coated BCB, as presented in Figure 1 and described in Section 2.2.

#### 3.2.1. Vibration Sensor with Horizontal Feed-Throughs

**Figure 4:**
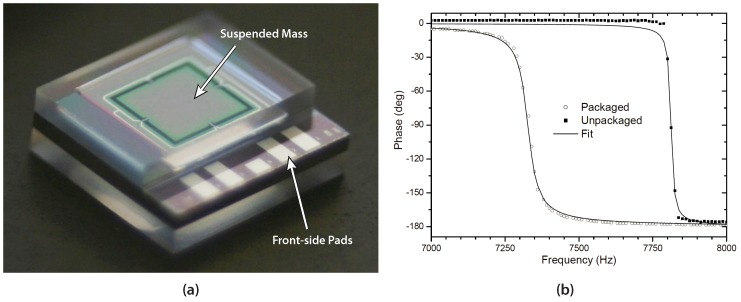
(**a**) Fully fabricated vibration sensor with front and back-side glass capping. The front-side pads were revealed by partial dicing of the capping substrate; (**b**) Phase response plot of an unpackaged and packaged vibration sensor. The phase response was fitted to a second order system given in Equation (1) to find the *Q*-factors and resonant frequencies. Originally published in Proceedings of the Pan Pacific Microelectronics Symposium, Kauai, HI, USA, 22–24 January 2008.

A MEMS vibration sensor for condition monitoring of industrial machines has been developed and demonstrated using the proposed capping method with horizontal feed-through interconnections, as illustrated in Figure 2d. The MEMS sensor itself is comprised of a rectangular mass suspended by four single-crystalline Si bridges with integrated piezo-resistive sensors. The device was packaged using both front and back-side capping, as shown in the process sequence depicted in Figure 2a–d. To provide a sufficient margin for the partial dicing, 60–80μm-deep cavities were etched into the capping substrates. For the bonding, a 3μm-thick spray-coated BCB adhesive layer was chosen to compensate for the ∼1 μm topography of the surface feed-through metal lines on the device substrate. The bonding was then performed inside the bond chamber under a gas pressure of 300mbar. Figure 4a shows a fully packaged and diced vibration sensor with exposed front-side pads. Note that the transparent lid allows for visual inspection of the suspended mass, even after encapsulation. A detailed description of the design and fabrication of the MEMS vibration sensor is presented in [25,27].

Electrical measurements of both packaged and unpackaged sensors were performed to evaluate the influence of the capping on the response function of the sensor. The packaged devices were glued and wire bonded to ceramic substrates, while the unpackaged devices were mounted on silicon spacers to allow the mass to move freely. The unpackaged sensors with silicon spacers were then glued and wire bonded to simple TO-cans (Transistor Outline Package) for mechanical testing. A shaker table was utilized to measure the transfer function of the packaged and unpackaged sensors.

The measurement results(shown in Figure 4b) reveal resonant frequencies of 7815Hz for an unpackaged sensor and 7327Hz for a packaged one. This slight reduction of resonant frequency suggests the introduction of a marginal amount of compressive stress, most likely due to the coefficient of thermal expansion (CTE) mismatch of glass and silicon. However, further analysis of nine vibration sensors confirmed that the average resonant frequency did not change considerably after the glass encapsulation, indicating that the capping process does not introduce significant tensile nor compressive stresses. Furthermore, the *Q*-factors were found by fitting the phase response to a second order system, described in Equation (1).

(1) $$\theta_{a}\left(\omega \right)=\theta_{0}+\arctan\left[-\frac{\omega \;\cdot \;\omega_{0}}{Q\;\cdot \;\left(\omega^{2}-{\omega_{0}}^{2}\right)}\right]$$

A reduction of the *Q*-factors from 750 for an unpackaged sensor down to 150 for a packaged sensor was detected. The reduced *Q*-factor of the packaged sensor indicates a moderate damping caused by the glass capping. Since the *Q*-factor reduction is caused by a damping effect, it does not affect the resonant frequency; this explains the discrepancy between the *Q*-factor reduction and the minute resonant frequency shift.

#### 3.2.2. MEMS Acceleration Switch with Vertical TSVs

**Figure 5:**
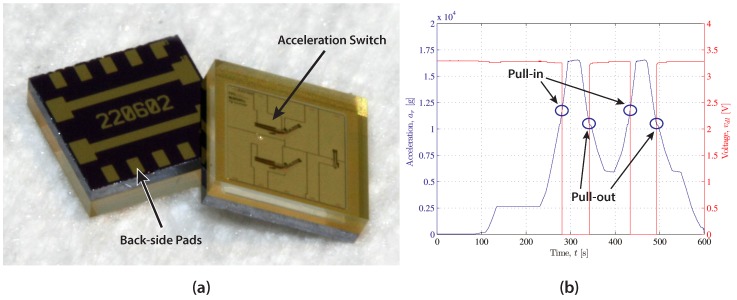
(**a**) Fully fabricated acceleration switch, showing the metal pads on the back-side (**left**) and the glass-encapsulated acceleration switch on the front-side (**right**); (**b**) Measurement result, displaying correct switching behavior with a pull-in acceleration of 11,800 *g* and a pull-out acceleration of 10,500 *g*. Originally distributed at the International Wafer-Level Packaging Conference, Santa Clara, CA, USA; 3–6 October 2011.

Acceleration switches are simple MEMS devices that open or close an electrical circuit if exposed to a certain acceleration threshold. Such an acceleration switch was designed, fabricated, and packaged by applying the proposed capping method in combination with TSV interconnections, as illustrated by the process sequence shown in Figure 2e–h. A via-first fabrication approach, in which the TSVs are fabricated before the MEMS structures, was employed to create heavily doped polysilicon vias with a footprint of 7 μm x 70 μm [22,28]. After the MEMS switch fabrication, the devices were packaged using the glass capping process, and metal pads were fabricated on the back side of the substrate. The depth of the cavities in the capping substrate was chosen to be 20μm, and a 1.4μm-thick spray-coated BCB adhesive layer was used. This comparably thin BCB layer was chosen because the corresponding bonding area on the device wafer surface did not feature any significant topography. The gas pressure inside the bond chamber was held at 500mbar during the bonding process. A finished acceleration switch is shown in Figure 5a, displaying both the metal pads on the back-side as well as the switches below the transparent lid on the front-side of the chip.

For the evaluation of the acceleration switches, the finished devices were flip-chip bonded to a test circuit using conductive glue that was stencil-printed onto the flip-chip pads. A Sorvall WX80 Ultra centrifuge (Thermo Fisher Scientific Inc., Waltham, MA, USA) was used to generate a very large acceleration for testing the switches. The measurement plot in Figure 5b displays a reliable pull-in of the acceleration switch at 11,800 *g* and a pull-out at 10,500 *g*. The observed hysteresis of the switch is most likely due to stiction forces at the switch contact. Our device evaluation confirms successful operation of the acceleration switch and the TSVs, as well as an intact bond frame that withstands the involved mechanical stresses and does not impede the functionality of the encapsulated device.

## 4. Conclusions

A simple and cost-effective wafer-level capping method for MEMS and imaging sensors has been presented in this paper. The key advantages of this method over alternative packaging approaches are that it does not require any surface processing or structural preparation on the device substrate, and that no patterning of the adhesive polymer is necessary. Therefore, non-photosensitive adhesives can be used, which lowers the process complexity and fabrication costs. A low bonding temperature of $250\;{}^{\circ }C$ ensures full compatibility for capping of CMOS wafers. The excellent mechanical and chemical stability of the bonding polymer enables a large variety of post-bonding fabrication processes. Additionally, the near-perfect optical transparency of BCB combined with the thinness of the deposited layer make this capping method very well suited for optical applications. The presented capping method was demonstrated by encapsulating MEMS vibration sensors and MEMS accelerations switches, using two separate approaches to create electrical interconnections from inside the cavity to the outside world. The experimental measurements confirm correct operation of both types of devices, integrated by both wire bonding and flip-chip bonding. Further analysis showed that the capping process does not introduce any significant mechanical stress to the devices and achieves a consistent bond strength of >20–30 MPa. Thus, the demonstrated capping method is a very attractive approach for low-cost encapsulation of a variety of devices, including MEMS and imaging sensors.

As possible future work, a more detailed study of the sealing ring geometry could be envisioned. Optimizing the sealing ring width and minimizing the necessary clearance to the encapsulated device structure would allow for smaller device footprints and higher integration density.

## Abbreviations

BCB	Benzocyclobutene
CMOS	Complementary metal-oxide-semiconductor
CTE	Coefficient of thermal expansion
IC	Integrated circuit
MEMS	Micro electro-mechanical systems
SEM	Scanning electron microscope
TSV	Through-silicon via
